# Gastric Volvulus: A Complication of Hiatal Hernia

**DOI:** 10.7759/cureus.11123

**Published:** 2020-10-24

**Authors:** Sara Lourenço, Ana Marta Pereira, Marta Guimarães, Mário Nora

**Affiliations:** 1 General Surgery, Centro Hospitalar de Entre Douro e Vouga, Santa Maria da Feira, PRT

**Keywords:** gastric volvulus, hiatal hernia

## Abstract

Gastric volvulus is a rare but potentially life-threatening condition, with difficult diagnosis. We present a case report of a demented woman aged 65 years that attended the emergency department with epigastric pain and vomiting for the past 10 days.

The chest plain revealed a retrocardiac air-filled mass and the abdomino-pelvic computed tomography confirmed the diagnosis of gastric volvulus. The patient was admitted. A nasogastric tube was introduced, antibiotics and parenteric nutrition were started and the patient didn’t eat anything. The patient was operated at fifth day of admission by laparoscopy. There weren’t signs of gastric necrosis, so the stomach was mobilized for its natural position on abdominal cavity, the hiatal defect was closed and a Nissen fundoplicature was performed. The post operative period was uneventful and the patient was discharged on the third post-operative day, without any complication.

This case illustrates a sub acute presentation form of gastric volvulus and a differed minimally invasive approach attending at the patient's clinical stability.

## Introduction

Gastric volvulus (GV) is a rare cause of abdominal pain and can be a life-threatening condition [1]. It results from rotation of the stomach or part of it by more than 180° creating a closed-loop obstruction. The most difficult aspect of diagnosing gastric volvulus is its consideration as part of differential diagnosis. Acute GV is associated with a high morbidity and mortality (30-50%), secondary to gastric ischaemia, perforation and necrosis [2].

## Case presentation

A 65-year-old woman presented in the emergency department with a 10 day history of epigastric pain and vomiting. She also had anorexia and weight loss. She denied history of regurgitation, heartburn, retrosternal pain or respiratory infections. On her medical background, she had arterial hypertension, dyslipidemia and dementia and was treated with perindopril, simvastatin, maprotiline, tiapride, zolpidem and alprazolam.

The patient was hemodynamically stable and the physical exam and blood analysis didn’t reveal alterations.

**Figure 1:**
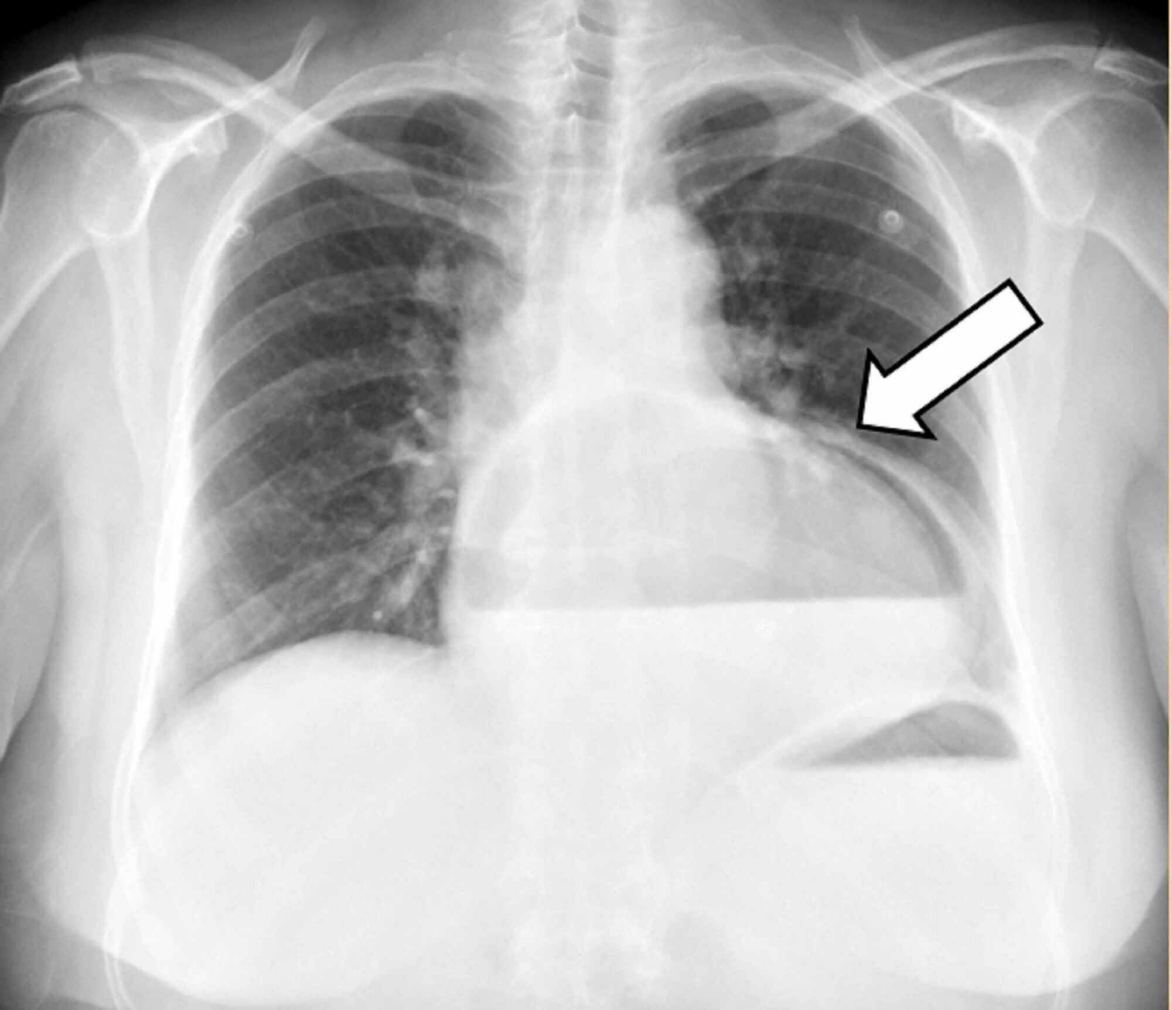
Chest radiograph showing a retrocardiac air-filled mass (white arrow)

The thoracic x-ray revealed a retrocardiac air-filled mass, suggestive of a huge hiatal hernia (Figure 1). A nasogastric tube was introduced, leaving a small amount of food content, with symptom improvement.

**Figure 2:**
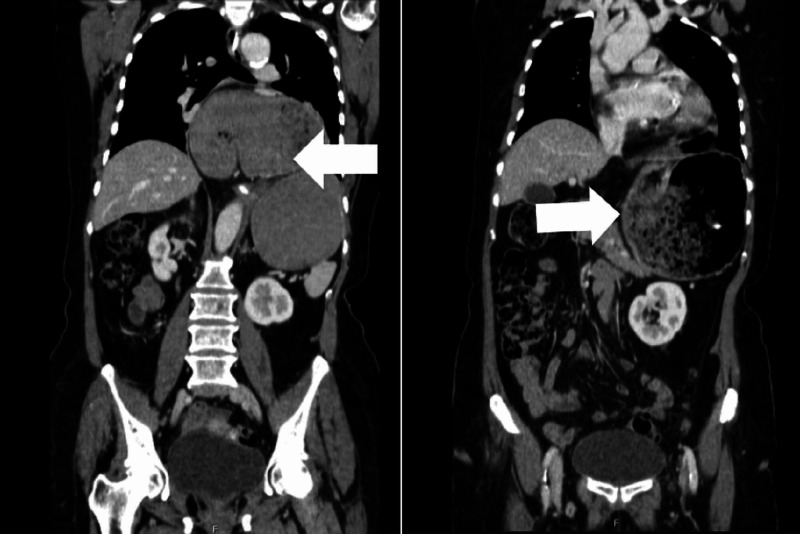
Abdomino-Pelvic tomography showing a markedly distended stomach (white arrow)

In the abdomino-pelvic tomography a markedly distended stomach was observed in a retrocardiac location, showing the gastric antrum in the posterior mediastinum. Concomitantly, the gastric walls were distended and hypodense, and there were signs of air in liver parenchyma suggestive of a mesenteroaxial gastric volvulus (Figure 2).

The patient was admitted and continuously monitored (heart rate, blood pressure, electrocardiography and peripheral oxygen saturation). During the hospital stay, the nasogastric tube was kept in free drainage, the patient was left fasting, a parenteral nutrition was infused to improve nutritional status and antibiotherapy was administered with cefuroxime and metronidazole due to the risk of bacterial translocation. She was always without fever, hemodynamically stable and without abdominal pain.

She was operated on the fifth day of hospital stay by laparoscopy. There were no signs of gastric necrosis, so the stomach was mobilized from the thorax to abdomen, the hiatal defect was closed and a Nissen-Rossetti fundoplicature was performed.

The postoperative period was uneventful, the patient tolerated progression in the diet and was discharged on the third postoperative day, without any complication, with indication for ingestion of liquid diet.

The patient was reassessed after one week and she did not present any symptoms, so she started to eat solid food. The patient was reassessed after three months and she was asymptomatic.

## Discussion

The term “volvulus” is derived from the Latin word “volvere”, which means “to roll or twist” [3].GV was described for the first time by Berti in 1866 and the first successful surgery of GV was performed by Berg in 1897 [4]. The incidence and prevalence of GV are unknown. The majority of cases are observed in the fifth decade of life, with no predilections for a particular gender or race [2].

Based on the aetiology, GV can be classified as primary or secondary. In 30% of patients, GV occurs as a primary event due to a malignancy, adhesions or failure of the gastric supports (gastrocolic, gastrosplenic, gastrophrenic and gastrohepatic ligaments). In the majority of cases, GV occurs secondary to a paraesophageal hernia, eventration of diaphragm, abdominal bands or adhesions and phrenic nerve paralysis [5].

There are four types of GV, according to the axis rotation. In type 1, or organoaxial rotation, the stomach rotates around the pylorus-cardia axis and this is the most common type, comprising about 60% of cases. In type 2, or mesenteroaxial rotation, the stomach rotates around the axis causing bisection of greater and lesser curvature of the stomach. Type 3 is a combination form of organoaxial and mesenteroaxial rotation, accounting for only 2% of cases. Type 4 is unclassified (occurs in 10% of cases) [6].

The clinical presentation of patients with GV depends on the speed of onset, the type of volvulus and the degree of obstruction. Classic symptoms of acute GV are epigastric pain, unproductive retching and the inability to pass a nasogastric tube. These symptoms are known as Borchardt’s triad and are present in 70% of cases [2]. GV can also be associated with hematemesis, nausea and hiccups. Haemorrhage occurs due mucosal injury as a result of ischemia or mucosal tear due to retching [7]. A volvulus may also present with chronic symptoms of dysphagia, postprandial pain, vomiting and breathlessness [8]. The complications of GV include perforation, peritonitis, shock and death, particularly when it is an acute GV [4].

Due to the nonspecific symptoms of GV, the diagnosis may be missed unless a high index of suspicion is maintained. Diagnosis is therefore difficult and it is conventionally achieved based upon a combination of history and imaging findings [9]. A chest radiograph demonstrates a retrocardiac, air-filled mass with an air-fluid level, whereas the abdominal films may reveal a distended fluid-filled stomach [2].

The abdominal computed tomography can confirm the diagnosis of GV, revealing anatomical defects, showing evidence of perforation, pneumatosis in the stomach wall and allows the exclusion of other problems [10].

As a result of vomiting, decreased oral intake and dehydration and electrolyte unbalance may be present [8].

Upper gastrointestinal endoscopy plays both a diagnostic and therapeutic role. It is often performed preoperatively to rule out an underlying gastric malignancy. However, in acute GV it can show mucosal congestion/ulceration, identify a bleeding source together with the inability to pass the scope through the pylorus. In the chronic GV, endoscopy can reduce the volvulus, although there are some reports in the literature revealing successful endoscopic reduction for acute GV. However, care should be taken not to cause perforation or, if a perforation is suspected, upper endoscopy should not be performed [11].

The management of GV depends on the degree of gastric obstruction, the presence of gastric ischemia and the comorbidities of the patient. Taking into account these factors, patients with mild obstructive symptoms may be managed electively, while patients with complete gastric obstruction, suspected of ischemia or perforation and hemodynamically unstable will require urgent treatment. The risk of gastric strangulation is greater with organoaxial volvulus and with severe gastric torsion (>180 degrees) and a delay in the treatment results in a mortality increase [12].

Treatment of GV has evolved over the years. The initial treatment approach for acute GV consists of resuscitation, placement of a nasogastric tube for gastric decompression and resting in the prone position [1,2].

The surgical approach is the treatment of choice of GV. The aim of surgery includes decompression of the stomach with reduction of the volvulus, gastropexy and correction of the intra-abdominal factors predisposing to volvulus. In the presence of several complications, such as gangrene or perforation, partial or total gastrectomy may be necessary [13]. Fifty years ago, Tanner described several operative steps: diaphragmatic hernia repair, simple gastropexy, gastropexy with division of the gastrocolic omentum (Tanner’s operation), partial gastrectomy, fundo-antral gastrogastrostomy (Opolzer’s operation) and repair of eventration of the diaphragm [14]. Classically this was performed by laparotomy, however laparoscopic approach has been shown to be safe and effective in selected patients and laparoscopy is associated with fast recovery, shorter hospital stay and lesser wound infections, among others [15].

In patients with high surgical risk, other less invasive techniques have been used [16]. Endoscopic treatment allows volvulus reduction and stomach fixation with a percutaneous endoscopic gastrostomy (PEG) tube. In some situations, when there is a successful endoscopic reduction, a laparoscopic approach may be done. This procedure allows a safe reduction of volvulus, placement of a gastrostomy tube and treatment of an underlying disease like hiatal hernia [17,18].

Quick recognition and prompt surgical approach remain the mainstays of therapy for acute GV. The reported overall mortality rates range from 30-50%, as a result of delayed recognition and treatment. Without early detection, the likelihood of strangulation increases, and this is the leading cause of the high mortality rate [19,20].

## Conclusions

GV is a rare entity with non-specific and vague symptoms with a high mortality rate. Clinical suspicion for GV should be maintained on initial presentation in the setting of retching, vomiting, epigastric pain and elderly age. So, a prompt diagnosis and appropriate treatment is mandatory to improve outcomes in these patients. The laparoscopic approach is safe and effective for GV treatment, since it was performed by experienced teams.

## Figures and Tables

**Figure 3:**
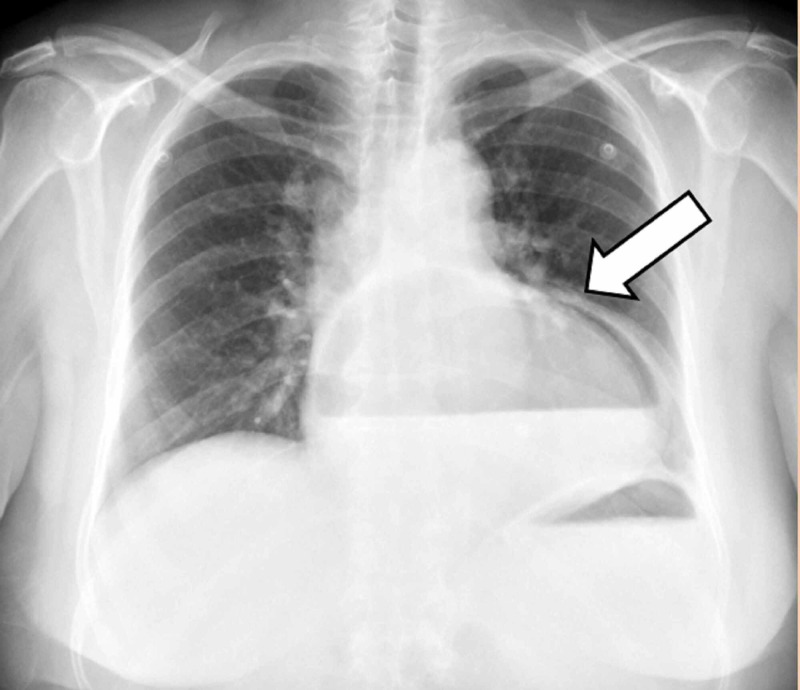
Chest radiograph showing a retrocardiac air-filled mass (white arrow)

**Figure 4:**
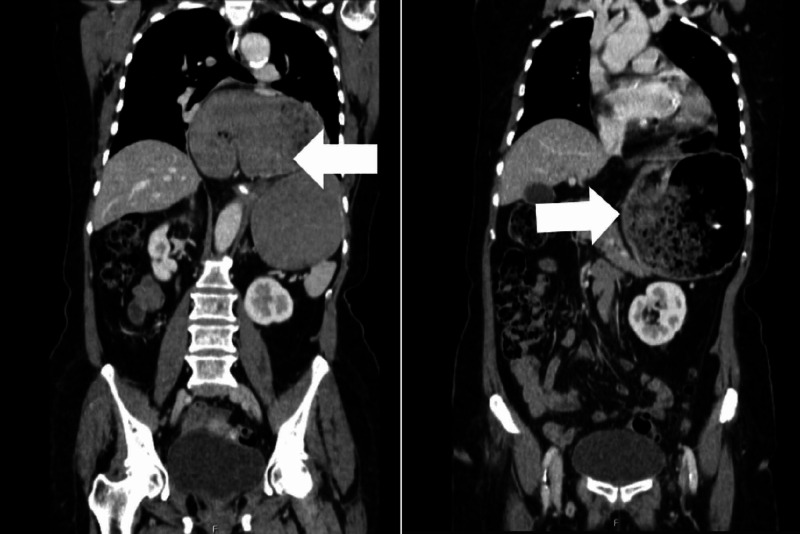
Abdomino-Pelvic tomography showing a markedly distended stomach (white arrow)
